# Immediate Effects and Experiences of a Digital Single-Session Behavioural Activation Based Intervention for Adolescents: A Single Arm Pre-post Programme Evaluation of Project ABC in the UK

**DOI:** 10.1177/13591045261433857

**Published:** 2026-03-19

**Authors:** Sara Munir, Grace Perry, Jeffrey Lambert, Maria E. Loades

**Affiliations:** 1 Department of Psychology, 1555 University of Bath, Bath, UK; 2 Department of Health, 1555 University of Bath, Bath, UK; 3 University of Bath Mental Health Research Group (MHRG), Bath, UK

**Keywords:** depression, adolescent mental health, scalable interventions, digital mental health intervention, early help, single session intervention, behavioural activation

## Abstract

**Background:**

Self-guided Digital Mental Health Interventions (DMHIs) are increasingly used amongst young people as they are scalable and may improve access to support.

**Objective:**

To assess the acceptability, feasibility, utility, and immediate effects of Project ABC-UK, a Single-Session Behavioural Activation DMHI for UK youth.

**Methods:**

A single group pre-post design was used. Participants (aged 13–18) completed demographics, as well as measures of hope, self-agency, hopelessness, and perceived control before and after the online intervention. It was completed anonymously, and participants gave feedback. T- and chi-square tests compared completers and non-completers; pre-post effects were assessed using paired t-tests with effect sizes (Cohen’s d). Thematic analysis explored feedback.

**Results:**

Of 799 participants, 401 (50.1%) completed the intervention, of whom 356 (88%) completed at least one post-intervention measure. Completers were more likely to be younger, have higher pre-intervention hope and self-agency, and to identify as sexual minorities. Significant improvements were found in hope and self-agency (*d* = −0.41), hopelessness (*d* = 0.52), and perceived control (*d* = 0.45). Most found the intervention enjoyable and useful.

**Conclusion:**

Findings support the feasibility and acceptability of Project ABC-UK, with promising immediate effects on all outcomes. Future studies should assess effects on depression and anxiety at follow-up and compared to other interventions.

## Plain Language Summary

Online mental health support can be useful for adolescents who find it difficult to access support from clinic based mental health services. Online mental health support can be made widely available on demand, meaning it could help adolescents when they need it, without waiting. It can also be made available anonymously, meaning they do not have to talk to adults to access it. We wanted to find out what UK adolescents (aged 13–18) thought about Project ABC, a self-help mental health support activity completed online, in one sitting. We asked adolescents to provide some demographic information about themselves, and to rate how hopeful/hopeless and in control of their life they felt before and after completing the Project ABC activity. They also provided some feedback on how they had found out about the activity, what they liked, and what could be improved or changed. In total, 799 adolescents agreed to take part in the activity, 401 completed the activity and 356 of them answered at least one question about feelings of hope/hopelessness and being in control of life after the activity. Younger adolescents (e.g., aged 13–15), adolescents who reported they had higher hope and control over their life before completing the activity, and sexual minority adolescents were more likely to complete the Project ABC activity. The activity also appeared to increase feelings of hope, and decrease feelings of hopelessness, and increase sense of control over life; adolescents also found it enjoyable and useful. To find out more about how the Project ABC activity compares to other options, future studies should compare it to other online mental health support activities, and also find out more about longer-term effects by including longer term follow up questionnaires.

## Background

Mental health disorders are the leading cause of disability and premature death globally among young people (McGorry, 2019). The COVID-19 pandemic, economic instability and climate change have contributed to the fragile mental health of the current generation of young people (Fusar-Poli et al., 2021). Globally, it is estimated that 14% of 10–19 year olds experience mental health disorders, with up to 5.5% and 3.5% of young people being diagnosed with anxiety and depression respectively, many of whom are left untreated (WHO, 2024), despite effective treatments being available. Numerous factors contribute to the needs-access gap including barriers to help-seeking like stigma, privacy concerns, lengthy waiting times and a lack of service provision (Garrido et al., 2019; Radez et al., 2021; Renton et al., 2014).

One potentially effective treatment for young people with depression symptoms is Behavioural Activation (BA) (Martin & Oliver, 2019; Tindall et al., 2024), and it is recommended by the National Institute for Health and Care Excellence (NICE, 2019). BA is based on the premise that a major contributor to the development and maintenance of low mood is withdrawing from enjoyable and meaningful activities. Therefore, BA aims to decrease inactivity by reintroducing personally meaningful, enjoyable, rewarding activities through monitoring activities and emotions, and activity scheduling (Loades & Essau, 2023).

Given that the need for help outweighs current available provision in young people’s mental health services, we need low-cost, scalable ways of providing evidence-based treatments like BA. Digital Mental Health Interventions (DMHIs) are delivered through mobile health applications, wearables, virtual reality systems, online/web-based platforms, chat bots or video games (Wies et al., 2021). By adapting evidence-based face to face treatments to digital delivery, these interventions can improve reach and access, and as such are critical in increasing mental health delivery for young people (Bevan Jones et al., 2020; Wangelin et al., 2016).

DMHIs may be a particularly useful solution for young people, who already use digital and online technologies as part of their daily lives. Young people find it helpful to seek help for their difficulties online due to immediacy, ease of access, inclusivity, and the sense of self-reliance of accessing DMHIs independently (Pretorius et al., 2019). In particular, the privacy and anonymity of self-guided DMHIs appeal to young people, as disclosing personal information does not involve the stress of face-to-face interactions (Higson-Sweeney et al., 2025; Wies et al., 2021).

DMHIs can also be effective in addressing mental health difficulties (Hollis et al., 2025). Systematic reviews have demonstrated that DMHIs have positive effects for symptoms of depression and anxiety in young people (Lattie et al., 2019; Lehtimaki et al., 2021; Pennant et al., 2015). However, a systematic review which looked at both guided and self-guided DMHIs found that digital interventions with a guided element (which may be from a parent, professional, peer or teacher) are more effective (Lehtimaki et al., 2021). Yet, many young people do not complete the full programme, with most finishing less than half of the recommended number of sessions (Jolstedt et al., 2018; March et al., 2018). Thus, intentionally designing DMHIs as stand-alone, one-off encounters could enhance adherence and hence effectiveness.

Single Session Interventions (SSIs) are brief, time-limited interventions that deliver the key concepts of an intervention in a single encounter (Schleider et al., 2025). SSIs transcend many existing barriers to traditional mental health provision such as stigma, and could address retention difficulties of longer-term DMHIs (Ludlow et al., 2023). Digital SSIs, including those that are self-guided, have small but significant positive effects for anxiety and depression in young people (Ball et al., 2024; Parlak et al., 2025). Additionally, an umbrella review of SSIs across all age groups found that 83% of reviews reported positive effects on one or more outcomes including anxiety, depression, externalising problems, eating disorders, substance use, and engagement (Schleider et al., 2025).

The Project Action Brings Change (A.B.C.) SSI is a Single Session self-guided BA Intervention, which was developed by Schleider and colleagues and trialled in the US for adolescents with internalising distress (Schleider et al., 2020). When tested in a randomised control trial in young people aged 13–16 in the USA, those allocated to Project ABC had reduced symptoms of depression and anxiety, hopelessness, and increased perceived agency at three-month follow up relative to a placebo control SSI and had comparable outcomes to the growth mindset based Project Personality SSI (Schleider et al., 2022). When offered as part of an open access platform study, Project ABC was selected by 37% of US-based young people aged 11–17 and showed a 37% completion rate, with significant reductions in hopelessness, perceived control, self-hate and agency immediately post intervention (Schleider et al., 2020).

Although the Project ABC SSI has shown promising results in both open platform evaluations and randomised control trial the US, it has not been evaluated in for adolescents in the UK context. In view of cultural and systemic differences in educational and healthcare settings between the two countries, we conducted a pilot study of Project ABC in the UK. We aimed to investigate the acceptability, feasibility and immediate effects of Project ABC. Based on the data available from studies on US adolescents, we anticipated that the Project ABC SSI would be acceptable, feasible, and show initial positive immediate effects in the UK context.

## Method

We conducted a pre-registered (https://osf.io/jmux5/overview) non-randomised, single-arm prospective study.

### Participants

Participants were UK based young people aged 13–18, recruited between March 2024 and January 2025. Project ABC-UK was advertised through various channels such as unpaid social media posts (Instagram and X, formerly Twitter), mental health organisations, and schools. Study adverts included basic study information and inclusion criteria and gave a weblink/QR code for interested potential participants to access more information. Participants who stated that they were older than 18, younger than 13, or were not comfortable speaking English, were excluded. Screen time data was used to detect and exclude bots (Falk et al., 2025).

We aimed to recruit at least 100 participants to attain statistical power of 0.80 (Kelley & Maxwell, 2012). Based on prior US based evaluations (Schleider et al., 2020), we predicted a small effect size (*d* = 0.3), *α* = 0.05.

### Procedures

All study procedures took place on the Qualtrics survey platform. The eligibility check involved asking participants their age, whether they lived in the UK, and whether they were comfortable speaking English. Those who were eligible were presented with a study information sheet. Participants aged 16 and above were directed to a consent form. Young people under the age of 16 could seek parental consent or opt to attempt to demonstrate Gillick competence (NHS, 2019) to consent for themselves by completing four multiple choice questions to demonstrate their understanding of the purpose of the study and what was involved, a consent procedure that was developed for low risk online, anonymous studies (Loades et al., 2025). If participants were not able to pass on first or second attempt, they needed parental consent to take part.

Participants provided demographic information (age, sex at birth, gender identity, sexuality), indicated where they heard about the study, and completed baseline measures (psychometric measures). Participants then completed the ABC-UK intervention, post-intervention measures, and provided feedback about their experience of the intervention.

### Project ABC Intervention

The Behavioural Activation based Project ABC SSI (Schleider et al., 2020) includes psychoeducation around the behaviour-emotion link, breaking the cycle of avoidance, taking action to reverse negative mood spirals, and setting and achieving specific values-based goals in a manageable way. The intervention includes interactive exercises to consolidate learning. For example, participants are asked to rate their mood after being shown a humorous video, to illustrate that what you do affects how you feel. Additionally, participants make a specific positive action plan and think about the ‘roadblocks’ to achieving goals. At the end, participants are provided with their self-made action plan. Throughout, participants are provided with vignettes of real-world examples of each concept to aid their understanding, and to access relatable experiences of other young people experiencing depression. This SSI takes around 15 to 20 min to complete and includes a read-aloud function for accessibility.

### Measures

The study measures are detailed in Table 1. All demographic measures and measures of clinical characteristics were completed before the intervention only, while proximal outcomes were completed before and after the intervention, and the intervention feedback was completed after the intervention only.

**Table 1.** Study Measures

Measure name	Authors	Measure type (what it measures)	Description
Demographic questionnaire	Loades et al. (2025)	Demographics	The demographic questionnaire (used in Loades et al., 2025) asked participants to provide information about where they found out about the study, their biological sex, gender, sexual orientation, and ethnic group. All questions were multiple choice, and participants could only provide one answer (except for gender identity, where multiple answers were permitted).
McArthur subjective social status scale – youth 2 item version	Goodman et al. (2001)	Subjective social status (part of the demographic questionnaire)	Participants completed the McArthur scale of subjective social status (Goodman et al., 2001). Subjective social status (SSS) refers to an individual’s perception of rank when compared with others, which is relevant to this study because lower SSS is associated with more depressive symptoms (Rahal et al., 2020). The McArthur scale adapted for adolescents is originally a 10-item measure (Goodman et al., 2001). We used a two-item measure which asks young people to rate their family’s social status, and their status in school, as compared to others, using a 10-rung ladder image. The top of the ladder represents the people that are ‘best off’ and the bottom of the ladder represents people who are ‘worst off’. Higher and lower scores indicate higher and lower perceived rank respectively (Karvonen & Rahkonen, 2011). This measure presents excellent reliability in young people (Goodman et al., 2001).
Patient health questionnaire – 2 (PHQ-2)	Kroenke et al. (2003)	Clinical characteristics (depression symptoms)	The PHQ-2 is a condensed version of the PHQ-9, consisting of two items designed for preliminary depression screening in adolescents (Allgaier et al., 2012), with scores >3 indicating depression. It examines frequency of low mood over the last two weeks, scoring from 0 (not at all) to 3 (nearly every day) (Kroenke et al., 2003). It has demonstrated strong diagnostic accuracy in identifying depression within this population (Richardson et al., 2010), as well as construct and criterion validity (Kroenke et al., 2003). In our survey, we included a third item which asked participants to rate how much their symptoms were causing them difficulty with daily tasks, from 0 (not difficult) to 4 (extremely difficult). This measure is scored out of 12, with higher scores indicating greater severity of depression.
Generalised anxiety disorder – 2 (GAD-2)	Kroenke et al. (2007)	Clinical characteristics (anxiety symptoms)	The GAD-2 is a reliable and valid measure of generalised anxiety symptoms, assessing how frequently symptoms have occurred over the last two weeks, rated from 0 (not at all) to 3 (nearly every day), with total scores >3 indicating GAD. The GAD-2 has shown construct and convergent reliability in young people (Byrd-Bredbenner et al., 2021). In our survey, we included a third item which asked participants to rate how much their symptoms were causing them difficulty with daily tasks, from 0 (not difficult) to 4 (extremely difficult). This measure is scored out of 12, with higher scores indicating severity of anxiety.
State hope scale (SHS)	Snyder et al. (1996)	Proximal outcome (perceived self-agency)	The SHS is a 6-item measure of goal-directed thinking and behaviour which requires participants to state from 1 (definitely false) to 8 (definitely true) how much they agree with a statement (Snyder et al., 1996). This measure demonstrates internal consistency, reliability and construct validity (Brooks & Hirsch, 2017). We used the “agency” subscale of this measure, which uses 3 of the items to measure perceived ability to work towards goals, with higher scores indicating greater levels of hope.
Beck hopelessness scale (BHS)	Beck et al. (1974)	Proximal outcome (perceived hopelessness)	The BHS-4 is a brief, four-item version of the Beck hopelessness scale, which has internal consistency and reliably measures hopelessness (Perczel Forintos et al., 2013). Participants describe their state of mind in the last week by rating how much they agree with a statement, from 1 (absolutely disagree) to 4 (absolutely agree), with a higher score indicating higher levels of hopelessness.
Perceived control	Schleider et al. (2020)	Proximal outcome (perceived sense of control)	A single-item measure of perceived control was created by Schleider and colleagues. Participants rated how much they agreed with the statement “right now, I feel like things are out of my control’” from 0 (not at all) to 10 (a lot) (Schleider et al., 2020).
Program feedback scale (PFS)	Schleider et al. (2020)	Intervention feedback	Post intervention, participants completed the program feedback scale (PFS), which comprises a 7-item Likert scale and three free-text boxes, assessing participants’ experience of the intervention (Schleider et al., 2020).

*Note.* This Table reports on the 11 measures used in this study, where the demographic questionnaire comprised of 4 unique demographic measures.

### Analysis

#### Patterns of Use and Sample Demographics

All data was analysed using SPSS, version 29.0.2.0 (20). To assess the utility of the SSI, we calculated figures and percentages of participants who had accessed various components of the study. Descriptive statistics (means, standard deviations and proportions) were calculated to summarise demographics and provide an overview of engagement.

To evaluate the differences in baseline demographics and psychometric variables between completers and non-completers of the intervention, we carried out independent samples t-tests and chi-square tests of independence. To improve validity of the chi-square tests, ethnicity and sexual orientation were recoded into broader categories, to reduce the number of cell counts below five.

#### Proximal Outcomes

We reported on mean scores and standard deviations of pre and post measures separately. To assess for statistically significant differences in proximal outcomes of the intervention, paired samples t-tests were conducted on pre- and post-intervention scores. In addition to statistical significance, effect sizes were calculated using Cohen’s *d* with 95% confidence intervals. Due to the large sample size (*N* >30), we followed the Central Limit Theorem (CLT) and assumed a normal sampling distribution (Field, 2018).

#### Intervention Feedback

We calculated item level percentages (%) of ratings given to each item on the program feedback scale. We completed inductive thematic analysis of the free-text feedback (Braun & Clarke, 2006). Based on Braun and Clarke’s phases of reflexive thematic analysis, we carried out data familiarisation and data coding of the first 100 feedback entries, generated initial themes, and applied these to the remaining participants, using constant comparison to assess whether new codes were required (Braun & Clarke, 2021). We used a relativist ontology informed by a constructionist approach to understand participants’ subjective experiences, which are shaped by contextual factors (Levers, 2013; Riger & Sigurvinsdottir, 2016).

#### Risks and Harms Monitoring

We also used pre-post measures to detect how many participants had reported a deterioration in proximal outcomes post intervention. Furthermore, we retrospectively searched the qualitative feedback from the PFS for any risks, harms and unintended consequences of the intervention (Lodewyk et al., 2023). We use the term Adverse ‘Event’ (AE), instead of ‘effect’, where there the unintended outcome may not be due to the intervention.

## Results

### Patterns of Use

**Figure 1.**
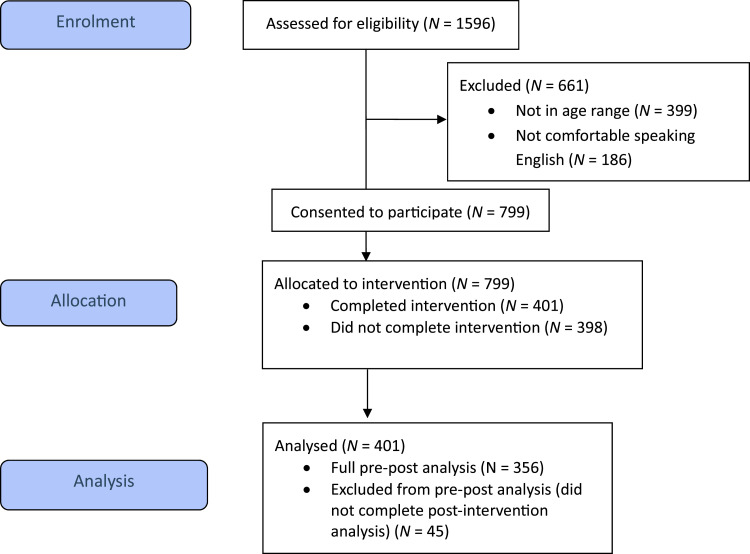
Participant flow *Note. N* pertains to the number of participants at each stage of the study

The participant flow is shown in Figure 1. Between March 2024 and January 2025, a total of 1,596 individuals accessed the Project ABC UK Qualtrics project. Of these, 799 young people consented to take part in the intervention. A total of 401 (50.1%) finished the intervention and of those, 88% (356/401) completed at least one post-intervention measure.

We compared the demographic and clinical characteristics of participants who completed the intervention to those who did not (see Table 2). Those who had completed the intervention were more likely to identify as sexual minority (*χ*^*2*^ (5, *N* = 685) = 24.68, *p* < .001). Completers also more likely to be younger (*t* (797) = 3.33, *p* < .001) and tended to have higher scores on the SHS which measures hope and self-agency (*t* (613) = −2.10, *p* = .036). Although completers mean scores GAD-2 and PHQ-2 scores were slightly lower scores than non-completers, the difference was not statistically significant.

**Table 2.** Demographic and Pre-intervention Clinical Characteristics of Completers and Non-completers

Variable	Completed (*N*), % OR M, SD	Not completed (*N*), % OR M, SD	Test statistic	*p*-value
Demographics				
Biological sex at birth			*χ*^*2*^*(*3) = 88	.277
Female	362 (90.3%)	253 (88.5%)		
Male	34 (8.5%)	29 (10.1%)		
Other/prefer not to say	5 (1.2%)	4 (14%)		
Ethnicity			*χ*^*2*^ (4) = 5.27	0.261
White/white other	309 (77.15)	203 (72%)		
Mixed	19 (4.7%)	21 (7.4%)		
Asian	53 (13.2%)	39 (13.8%)		
Black	12 (3%)	15 (5.3%)		
Other	8 (2%)	4 (1%)		
Sexuality			*χ*^*2*^*(*5) = 24.68	<0.001
Heterosexual	160 (39.9%)	148 (52.1%)		
Homosexual	52 (13%)	20 (7%)		
Bisexual	63 (15.7%)	49 (17.3)		
Pansexual, asexual, queer	35 (8.7%)	15 (5.3%)		
Unsure, no label, other	90 (22.24%)	45 (15.8%)		
Prefer not to say	1 (0.2%)	7 (2.5%)		
Age	15.51, 157	15.88, 1.57	*t* (797) = 3.33	<0.001
Psychometrics				
GAD-2	8.95, 2.02	9.30, 2.02	*t* (311) = 1.483	0.139
PHQ-2	8.27, 2.30	8.34, 2.34	*t* (652) = 0.38	0.706
SHS	12.47, 4.66	11.67, 4.26	*t* (613) = −2.10	0.36
BHS	11.11, 3.21	11.56, 3.20	*t* (609) = 1.66	0.98
Control	7.77, 2.42	7.91, 2.52	*t* (612) = 0.65	0.515
McArthur social status question 1	5.19, 1.57	5.14, 1.86	*t* (518.4) = −0.33	0.745
McArthur social status question 2	5.17, 2.09	5.31, 2.26	*t* (668) = 0.86	0.396

*Note.* GAD-2: Generalised Anxiety Disorder- 2, PHQ-2: Patient Health Questionnaire- 2, SHS: State Hope Scale, BHS: Beck Hopelessness Scale.

### Sample Characteristics

The mean age of participants was 15.7 (SD = 1.6), with most young people identifying as female (89.5%), and white British (74.9%). Over half of our participants (53.9%) identified as sexual minority. See Table 3 for details.

**Table 3.** Baseline Demographics and Clinical Characteristics

Participant characteristics	Intervention (*n* = 799) *N* (%)
Age (years), mean (SD)	15.7 (1.6)
Age 13–14	204 (25.6%)
15–16	293 (36.7%)
17–18	302 (37.8%)
Sex recorded at birth (female), *n* (%)	615 (89.5%)
Sex recorded at birth (male), *n* (%)	63 (9.2%)
Prefer not to say	7 (1%)
Sexual orientation, *n* (%)	*N* = 685
Heterosexual	308 (45%)
Bisexual	112 (16.4%)
Homosexual	72 (10.5%)
Other (pansexual, queer, asexual or other/not listed)	78 (11.4%)
Unsure/do not use a label	107 (15.6%)
Do not want to respond	8 (1.2%)
Missing	114
Ethnicity, *n* (%)	*N* = 683
White or White British	512 (74.9%)
Asian or Asian British	92 (13.5%)
Black or Black British	27 (3.9%)
Mixed (white and black Caribbean, white and black African, white and Asian, any other mixed)	40 (5.9%)
Arab	6 (.9%)
Other	6 (.9%)
Missing	116

Pre-intervention, mean depression symptom severity (PHQ-2) scores were 8.32 (SD = 2.25, range = 3–12), with a mean anxiety symptom severity (GAD-2) score of 9.18 (SD = 2.02, range = 4–12), indicating high levels of depression and anxiety symptom severity among this sample. On the McArthur Scale questions, the mean score was around 5, with participants using the full range of scores (1–10 on both questions), indicating that our sample came from across the social status spectrum. Mean SHS scores were 12.26 (SD = 4.57, range = 3–24), with moderate levels of hope and self-agency and the BHS had a mean of 11.43 (SD = 3.02, range = 4–14), indicative of high levels of hopelessness. Additionally, mean perceived control was 7.8 (SD = 2.38, range = 0–10), indicating that most participants felt a perceived lack of control prior to starting the intervention.

### Exploratory Pre-Post Intervention Outcomes Analysis

Analysis revealed a statistically significant change in State Hope Scale scores, which increased from pre-intervention (M = 12.26, SD = 4.57) to post-intervention (M = 13.93, SD = 5.21), *t* (258) = −6.59, *p* < 0.001, indicating significant increases in hope. The effect size was moderate (*d* = −0.41, 95% *CI* [−0.53, −0.28]).

There was a statistically significant decrease in Beck Hopelessness Scale scores from pre intervention (M = 11.43, SD = 3.02), to post intervention (M = 10.36, SD = 3.22), *t* (253) = 8.25, *p* < 0.001, indicating a statistically significant decrease in hopelessness. The effect size was moderate (*d* = 0.52, 95% *CI* [0.38–0.64].

Results showed a decrease in the single item Perceived Control measure, from pre intervention (M = 7.82, SD = 2.38) to post intervention (M = 7.02, SD = 2.54), *t* (257) = 7.22, *p* < 0.001, demonstrating a statistically significant decrease in young people feeling out of control, post intervention. The effect size was moderate (*d* = 0.45, 95% *CI* [0.32–0.57]).

See Table 4 for within group differences and proximal outcomes.

**Table 4.** Within-Group Differences in Proximal Mental Health Outcomes Among Young People taking Part in Project ABC-UK

Outcome	Baseline M (SD), range	Post-intervention M (SD), range	*p*-value	95 % *CI*
PHQ-2	8.34 (2.25) range = 3–12			
GAD-2	9.18 (2.02) range = 4–12			
MacArthur 1 (socioeconomic status in UK)	5.17 (1.7) range = 1–10			
McArthur 2 (socioeconomic status in school)	5.2 (2.2) range = 1–10			
SHS	12.26 (4.57) range = 3–24	13.95 (5.21) range = 3–24	<0.001	−0.53, −0.28
BHS	11.43 (3.02) range = 4–14	10.36 (3.22) range = 4–16	<0.001	0.38–0.64
Perceived control	7.82 (2.38) range = 0–10	7.02 (2.54) range = 0–10	<0.001	0.32–0.57

### Intervention Acceptability

**Figure 2.**
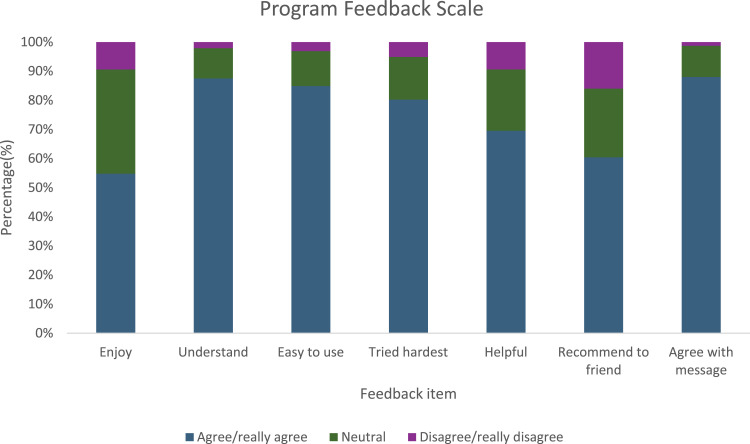
Program feedback scale: Frequency of participant ratings of intervention acceptability

Many participants who completed the Program Feedback Scale (*n* = 393) (see Figure 2 for results of the PFS) stated that they enjoyed the intervention (54.8%), agreed with its message (88.1%), understood the intervention (87.5%) and found it easy to use (84.9%). This shows that young people perceived the intervention to be highly accessible. Most young people tried their hardest while completing the intervention (80.2%), found the intervention helpful (69.6%), and reported that they would recommend the intervention to a friend (60.4%).

The free text feedback of participant’s experiences of Project ABC-UK resulted in the formulation of five prevailing themes: usability/accessibility, practical takeaways, length of intervention, depth of intervention, and improvements for action planning.

Participants articulated that the intervention was easy to use and tailored to individual needs. It was felt that the interactive aspect was helpful, and the read-aloud function increased accessibility of the intervention. Participants felt that the intervention left them with actionable advice, enabled them to set goals effectively, and the action plan feature was perceived to be particularly useful. Participants also expressed that the intervention helped them to alter their unhelpful ways of thinking. Additionally, participants described areas for improvement, such as the amount of reading required, and that fewer example scenarios would be preferable. Participants also felt that the intervention oversimplified their difficulties, and they would have appreciated delving deeper into their problems by reflecting on them during the intervention. Participants also would have preferred the plan to be clearer and more engaging, with a wider range of activities to integrate into their action plan. In free text answers in the PFS, 3 (0.8%) participants reported that the content of the intervention was upsetting, and 4 (1.1%) that the intervention was unhelpful. Additionally, 2 (0.56%) mentioned self-harm/suicidal ideation, although there was no suggestion that this was during or due to the intervention.

## Discussion

Our pilot of the American Project ABC SSI found that it was feasible, acceptable and potentially useful to young people in the UK. Specifically, young people who completed the SSI found it generally helpful and easy to use, and reported pre-post improvements in hope, perceived control and self-agency and decreased hopelessness. Therefore, Project ABC’s promising results in the US may be transferrable to a UK population of young people.

Approximately half of the young people who consented completed the intervention, which aligns with other similar studies (Clarke et al., 2015; Torous et al., 2020). Rates of intervention completion in Project ABC-UK compare favourably to some other DMHI studies where completion rates are closer to one third (Lattie et al., 2016; Lehtimaki et al., 2021; Ludlow et al., 2023; Schleider et al., 2020; Waumans et al., 2023). This may be due to the brief, standalone SSI format. Additionally, recruitment through mental health organisations may have attracted participants already motivated and engaged in help-seeking.

Project ABC-UK was mostly accessed by female participants (89.5%) which mirrors the demographic patterns of in person access to therapy (Kauer et al., 2014; Navarro et al., 2019). Additionally, similar to US-based SSI studies (McDanal et al., 2022; Schleider et al., 2020), around half our participants identified as sexual minority (53.9%), indicating that it may be a good way to reach this demographic group. Young people who identify as LGBTQ + particularly value digital privacy and confidentiality and may prefer to conceal their identity (Isbăşoiu et al., 2024; Lucassen et al., 2018). Therefore, the anonymity of self-guided DMHIs may particularly appeal to them, which is important given that they are both more vulnerable to developing mental health problems and also less likely to access traditional mental health services (Cai et al., 2024; Cohen & Schleider, 2022; Lucassen et al., 2018). Younger participants were also more likely to finish the intervention, which aligns with findings from Project YES (Schleider et al., 2020), and highlights a need to explore older adolescents’ experiences of these interventions and tailor them to better meet the needs of this demographic.

Consistent with US studies of the Project ABC SSI in adolescents (Schleider et al., 2020, 2021; Shroff et al., 2023), we found that perceived control, hope and self-agency increased and hopelessness decreased pre-post intervention. This is significant, as higher levels of hope and particularly the self-agency aspect of hope, are associated with lower levels of depression and anxiety, and greater perceptions of control are associated with lowered levels of depression (Myles & Merlo, 2022; Ritschel & Sheppard, 2018). Findings around hope and self-agency may be explained by hope theory, which posits that perceiving goals as attainable, a central feature of this intervention, results in investment in treatment and an increase in hope (Snyder & Taylor, 2000). This may also explain our findings that higher pre-intervention hope and self-agency scores were associated with completing the intervention.

Both the numerical feedback scale ratings and the qualitative feedback from free text boxes indicated strong acceptability, utility and relevance to young people in the UK. Participants particularly appreciated the actionable advice as well as goal setting and creating an action plan. Existing research has also found that young people value goal-setting and identifying future ambitions (Aschbrenner et al., 2019), which has been linked to a greater likelihood of academic attainment, general achievement, and self-efficacy (Pawlak & Moustafa, 2023) and hope (Penno et al., 2022). Participants’ feedback highlighted that they enjoyed the interactive aspect and that text-based content may have been excessive, consistent with previous research which has found that interactivity (e.g. tasks and quizzes) improves engagement (Gladstone et al., 2015; Jackson et al., 2024). Participants’ feedback mirrored previous young people’s DMHI research, which also found that interventions can be perceived as being too simplistic, broad and insufficient to address their difficulties (Higson-Sweeney et al., 2025; Jackson et al., 2024). Whilst young people may hope to explore their difficulties in greater depth, it is vital to maintain psychological safety for young people during self-guided DMHIs, particularly when there is a lack of clinician involvement (Allan et al., 2024).

### Strengths and Limitations

Project ABC-UK was publicly accessible; therefore, participation was self-initiated, resulting in a more naturalistic sample. We captured both quantitative outcomes and qualitative feedback, offering a well-rounded understanding of participants’ experiences.

We aimed to recruit a diverse sample by recruiting partially through organisations for under-represented communities and 74.9% of participants were White. This compares favourably to the general population in the UK (81.7% White, Census, 2021). However, 89.5% of participants were female, which limits the generalisability of our findings to males. Furthermore, due to errors with our data we were able to report on sex assigned at birth, but not on gender identity. Had we been able to report on this, it may have provided interesting and potentially important insights about the acceptability of the SSI in these groups given the growing need for gender-affirming psychological support for gender-diverse youths (Huit et al., 2024). We also did not measure geographic location or education level as part of this study, and acknowledge that exclusion of this contextual information limits the inferences we can make.

Additionally, we aimed to understand the immediate effects of the intervention. Future studies should include longer-term follow-up to evaluate the enduring effects of the intervention, including on depression symptoms. This is particularly pertinent where behavioural activation is concerned, as the positive impacts are generally observed beyond treatment (Busch et al., 2010). Future studies should also compare the Project ABC SSI to other interventions.

### Clinical Implications

Our findings support the use of brief, digital self-help interventions to provide scalable, accessible and anonymous support, including reaching young people who are under-served by traditional clinic-based support, such as those who identify as sexual minorities. SSIs like Project ABC may be useful as a widely available resource for emerging mental health difficulties in young people, particularly in community, primary care or educational settings, where young people are struggling to access evidence-based support.

Although proximal outcome measures indicated overall positive outcomes, a small number of participants reported adverse events, albeit not necessarily linked to the intervention itself. Although outcomes are often not analysed until data collection ends, qualitative feedback (where participants are likely to report AE) should be monitored regularly for timely management of any AE reported. Future SSIs could also consider additional risk measures such as offering check-ins for participants with higher pre-intervention scores or excluding those with active suicidal intent who may be better helped by emergency services.

### Conclusions

The current study supports the acceptability and feasibility of the Project ABC SSI for UK young people. Findings highlight the positive effects on hope, self- agency, hopelessness, and perceived control. Uptake and acceptability was particularly good amongst sexual minority young people, which highlights the need to offer anonymous digital interventions for this population.

## Data Availability

Anonymised study data can be made available upon reasonable request.*
